# Neoadjuvant Therapy for Neuroendocrine Neoplasms: Recent Progresses and Future Approaches

**DOI:** 10.3389/fendo.2021.651438

**Published:** 2021-07-26

**Authors:** Andrea Lania, Francesco Ferraù, Manila Rubino, Roberta Modica, Annamaria Colao, Antongiulio Faggiano

**Affiliations:** ^1^ Endocrinology, Diabetology and Andrology Unit, Humanitas Clinical and Research Center—IRCCS, Rozzano, Italy; ^2^ Department of Biomedical Sciences, Humanitas University, Rozzano, Italy; ^3^ Department of Human Pathology of Adulthood and Childhood ‘G. Barresi’, University of Messina, Messina, Italy; ^4^ Endocrine Unit, University Hospital G. Martino, Messina, Italy; ^5^ Division of Gastrointestinal Medical Oncology and Neuroendocrine Tumors, European Institute of Oncology, IEO, IRCCS, Milan, Italy; ^6^ Endocrinology, Department of Clinical Medicine and Surgery, “Federico II” University of Napoli, Napoli, Italy; ^7^ Endocrinology, Department of Experimental Medicine, “Sapienza”, University of Rome, Rome, Italy; ^8^ Department of Experimental Medicine, Division of Medical Physiopathology, Sapienza University of Rome, Rome, Italy

**Keywords:** neuroendocrine tumors, somatostatin (analogs and derivatives), peptide receptor radionuclide therapy, everolimus, capecitabine, temozolomide, chemotherapy

## Abstract

Neuroendocrine neoplasms (NENs) are a heterogeneous group of tumors, their treatment being challenging and requiring a multidisciplinary approach. Though the only curative treatment is surgery, up to 50% of patients are diagnosed with metastatic disease. In the last years, neoadjuvant chemo(radio)therapy has become part of the standard of care in the treatment of different cancer types. However, evidence of its efficacy and safety in NEN patients has not yet been confirmed in the literature. The aim of the present review is to perform an extensive review of the scientific evidence for neoadjuvant therapy in patients with gastroenteropancreatic and thoracic NENs.

## Introduction

Although neuroendocrine neoplasms (NENs) are considered rare malignancies, their incidence has rapidly increased in the last decades. Since several patients are diagnosed with metastatic disease, curative surgery is usually not an option (1), palliative surgical intervention possibly being effective in controlling clinical symptoms and improving patient’s quality of life (2–4). Neoadjuvant therapy, with the aim of reducing tumor size and disease burden, can potentially change the clinical scenario making it suitable for curative surgery as already demonstrated in other cancer types (5–8). While it is conceivable that neoadjuvant chemo-and radiotherapy might be effective in NENs (9), reliable evidence is still lacking in this field and study results are difficult to compare due the heterogeneity of both neoadjuvant therapies used, and series studied. Moreover, although the available therapeutic options (i.e., somatostatin analogs, everolimus, chemotherapy, sunitinib, and peptide receptor radionuclide therapy PRRT) are currently not included in any therapeutic algorithm with specific neoadjuvant purpose, most of them have been used with this intent, even successfully (9). Neoadjuvant chemotherapy, radiotherapy, and PRRT have been shown to provide variable results in terms of tumor down-sizing (10). The aim of the present minireview is to perform an extensive review of the scientific evidence for neoadjuvant therapy in patients with pancreatic, gastrointestinal, and thoracic NENs.

## Pancreatic Neuroendocrine Neoplasms

Pancreatic neuroendocrine neoplasms (pNENs) account for 1 to 2% of all pancreatic tumors and most of them are sporadic and nonfunctioning. Their incidence has been increasing and survival rates, although improved, remain poor compared with other primary sites, with an overall survival of 3.6 years (1). Often pNENs present with advanced disease at diagnosis and the treatment of metastatic unresectable pNENs remains debated and the role of neoadjuvant therapies is still uncertain. However, among gastrointestinal NENs, data on the possible role of neoadjuvant therapy are mainly related to pNENs even if the results seem to be somewhat contradictory and difficult to interpret due to the extreme heterogeneity of the data and the incompleteness of the information provided (**Table 1**). In a large series of high-grade gastrointestinal neuroendocrine carcinomas, where pancreas was the most common primary site (361 patients), neoadjuvant or adjuvant therapy resulted in better overall survival (OS) in patients with early-stage disease compared with those treated with resection alone. Details of neoadjuvant therapies were not available, but single and multi-agent chemotherapy besides radiotherapy were included. The positive effect of neoadjuvant or adjuvant therapy on OS seems to suggest the importance of these treatments to lower the incidence of both micrometastases and possibly to enhance tumor resection thus lowering the risk of local and systemic recurrences (26).

**Table 1:** Neoadjuvant therapies in gastroenteropancreatic NENs.

Year	Article type	Neoadjuvant treatment	Outcome	Reference
Pancreas				
2019	research article	no details available	no changes in OS	(11)
2008	research article	PRRT	Partial response and R0 resection	(12)
2009	case report	PRRT	Partial response and R0 resection	(13)
2010	case report	PRRT	Partial response and R0 resection	(14)
2011	research article	PRRT	Stabilization or partial response	(15)
2012	case report	PRRT	Partial response and R0 resection	(16)
2015	research article	PRRT	Better PFS and lower morbidity	(17)
2015	research article	PRRT	Improved PFS	(17)
2017	research article	PRRT	Improved PFS	(18)
2018	case report	PRRT	Partial response and R0 resection	(19)
2015	research article	chemo	Improved survival	(20)
2016	research article	chemo	Improved OS	(21)
2017	research article	chemo	no changes in tumor size	(22)
2018	research article	chemo	Improved OS and RFS	(23)
2011	case report	chemo, radio	R0 resection	(24)
2017	research article	chemo, radio	Stabilization or partial response and R0 resection	(25)
2018	research article	chemo, radio	Low recurrence risk and improved OS	(26)
2012	research article	chemo, radio	No effects	(27)
2012	research article	PRRT, chemo	Radiological response	(28)
2020	research article	PRRT, chemo	Stabilization or partial response	(29)
2020	research article	PRRT, chemo	Improved PFS and OS	(30)
Esophagous				
2018	case report	chemo	complete response, complete resection	(31)
1989	case report/review	chemo	almost complete response, complete resection	(32)
1995	case report	chemo	almost complete response, complete resection	(33)
1999	case report	chemo	almost complete response, complete resection	(34)
2002	case report	chemo	partial response, reduced tumor burden	(35)
2003	case report	chemo	partial response, reduced tumor burden	(3)
Rectum				
2017	research article	chemo, radio	almost complete response, complete resection	(36)
2018	case report	chemo	reduction of primary lesion/grading	(4)
Miscellanea				
2009	research article (midgut tumor)	PRRT	partial response, partial resection	(15)
2012	research article (midgut tumor)	PRRT	partial response, complete resection	(37)
2012	research article (midgut tumor)	SSA-PRRT	partial response, partial resection	(38)
2012	research article (duodenal tumor)	PRRT	partial response	(29)
2015	case report (small bowel tumor)	PRRT	no response	(39)

Chemo, chemotherapy; Radio, radiotherapy; PRRT, peptide receptor radionuclide therapy; SSA, somatostatin analogs; OS, overall survival; R0, microscopically margin-negative resection; PFS, progression-free survival; RFS, relapse free survival.

Patients who received neoadjuvant or adjuvant therapy had better over-all survival, suggesting that high incidence of micrometasta-sis contributes to the poor surgical outcomes. In addition, the relatively high proportion of margin-positive re-section raises the question of whether there is a role of downstaging with neoadjuvant therapy, aimed at enhancing resection and lowering risk of systemic recurrence.

Xie et al. recently described the largest group of pNENs in whom neoadjuvant therapies were used. The study included 4,892 patients who underwent curative-intent surgical resection. Authors showed that neoadjuvant therapy was mainly prescribed in patients <65 years, with grade 3 pNENs localized in the head of the pancreas and associated to the presence of metastasis. In this setting, Authors did not find any significant improvement in OS even in patients with grade 1 and 2 pNENs thus suggesting that neoadjuvant therapy should be used with caution given the lack of conclusive data (11). It is worth noting that, the main limitation of the Study was the lack of any detail regarding the type of neoadjuvant therapies used.

PRRT with radiolabeled somatostatin analogues, over the years, has evolved as an important therapeutic option for the treatment of inoperable or metastasized, well/moderately differentiated, NETs, particularly of the GEP (**Table 1**) (9). Conversely, neoadjuvant PRRT based on either 177Lu-octreotate or 90Y-DOTATATE have been shown to provide variable results as tumor downsizing possibly due to the heterogeneous inclusion criteria, the variable length of follow-up and the different response criteria used (10). Kwekkeboom et al. described a series of 310 patients with pNENs treated with 177Lu-octreotate PRRT. A partial response was observed in four of them, all undergoing a subsequent R0 resection (12). Similar results were confirmed by other case reports and small series (13, 14, 16, 19). Van Vliet et al. described the results of 177Lu-octreotate as neoadjuvant therapy in 29 patients with borderline, unresectable or oligometastatic nonfunctioning pNENs (14). After PRRT, successful surgery was performed in nine patients and all resection specimens showed fibrosis/sclerosis or necrosis thus confirming the effects of 177Lu-octreotate on tumor tissues. As a result, the median PFS was 69 months for patients with successful surgery and 49 months for the other patients (14). On the same line, other case reports and case series confirmed a beneficial effect of neoadjuvant PRRT with 90Y-DOTATATE on tumor and/or metastases downsizing in patients with pNENs, thus leading to a successful surgical intervention (15, 17). Recently, Partelli et al. reported a series of 23 resectable or potentially resectable G1-G2 pNENs who underwent neoadjuvant PRRT (177Lu-octreotate in three and 90Y-DOTATATE in 20 patients) compared to 23 patients who underwent upfront surgical operation. PRRT did result in a reduction of primary tumor size and a reduction in the number of positive lymph nodes compared with controls. Interestingly, though both the rates of disease-specific survival and median PFS from time of diagnosis were similar between groups, in the subgroup of patients who underwent an R0 resection, a trend toward a prolonged PFS was observed in the PRRT group (18).

The administration of chemotherapy prior to surgical resection is increasingly used for patients with adenocarcinomas of the pancreas with the aim to improve surgical results. Similarly, different regimens of chemotherapies have been described as neoadjuvant treatment in pNENs, all these studies being biased by their retrospective design and by the heterogeneity of chemotherapy regimens used (**Table 1**). Dumont et al. evaluated the effect of different neoadjuvant chemotherapy regimens (i.e., 5-fluorouracil, streptozotocin, doxorubicin, cisplatin, etoposide, and oxaliplatine) on segmental portal hypertension (SHP), the feasibility of surgery and the prognostic influence of a complete surgery in 42 patients with G1/G2 locally advanced pNETs associated to SHP. A complete resection was achieved in 13 out of 28 cases underwent surgery and a not statistically significant trend towards improved 5-year survival was observed in patients with R0 resections compared to R1/R2 resections and no resection at all (20). A retrospective analysis of 59 patients with a histologic diagnosis of pancreatic neuroendocrine carcinomas (pNECs) described five patients who underwent neoadjuvant treatment with etoposide and cisplatin before surgery. Four of them had a curative resection and one patient with stage IV disease remained with residual small liver metastases (21). A further retrospective observational study analyzed the efficacy of neoadjuvant 5-fluorouracil (5-FU), doxorubicin, and streptozocin (FAS) chemotherapy in 29 patients with non-metastatic locally advanced well-differentiated pNENs. In this series, neoadjuvant FAS did not induce a clinically significant change in the size of the primary tumor in up to 90% of treated patients thus suggesting that localized disease does not benefit from this preoperative treatment in terms of tumor downstaging (22). Preoperative FAS treatment has been further evaluated in a retrospective series of 27 patients with pancreatic neuroendocrine liver metastases (NELM) who underwent liver resection. Despite being associated with higher rates of synchronous disease, lymph node metastases, and larger tumor size, patients who underwent preoperative FAS had similar overall survival OS and RFS as patients who did not. Similarly, in patients who presented with synchronous liver metastases the median OS and RFS were significantly greater among patients who received preoperative FAS. Authors concluded that preoperative FAS could be considered for patients with advanced synchronous pancreatic NELM (23).

Few data are available on the effects of association of chemotherapy and radiotherapy as neoadjuvant therapy in pNENs, all these data being mainly obtained from case reports (**Table 1**). A poorly differentiated pNEC metastatic to the breast and lung was successfully managed with neoadjuvant chemotherapy (5-FU treatment followed by carboplatin and etoposide) and radiotherapy, followed by radical surgical resection (24). Among 33 patients with pNENs undergoing surgical resection with curative intent, 16 underwent surgery alone, while 17 underwent adjuvant or neoadjuvant external beam radiotherapy in addition to surgery. Fluoropyrimidine-based chemotherapy was delivered concurrently in 14 patients receiving radiotherapy. Local control in patients receiving combined modality therapy was like those who had surgery alone. Although the Authors conclude that the role of neoadjuvant radiotherapy remains unclear, it has been hypothesized that patients who underwent radiotherapy had more aggressive or extensive disease than the surgery alone group thus explaining the lack of any significant effect of combined neoadjuvant treatments (27).

Capecitabine combined with temozolomide (CAPTEM) has been frequently used in the treatment of pNENs. In particular, Strosberg et al. demonstrated that CAPTEM regimen was extremely effective for treatment of metastatic pNENs, resulting in an objective response rate of 70% and median PFS of 18 months (40). These data have been strengthened by a recent metanalysis confirming that capecitabine combined with temozolomide is effective for treating patients with advanced NENs, disease control rate being 72.8% (41). Neoadjuvant CAPTEM regimen with or without radiation has been successfully applied in six pNENs with borderline resectable disease. All patients had radiological evidence of tumor regression after neoadjuvant treatment (two partial responses and four stabilization) and all of them could undergo successful resection of the primary tumor with negative margins in 4/6 patients (25). The efficacy of CAPTEM regimen in the neoadjuvant setting was further confirmed by Ostwal et al. who studied 30 patients with locally advanced pNENs or pancreatic neuroendocrine hepatic metastases receiving neoadjuvant CAPTEM. Partial response was observed in 13 of them, while a stable disease was found in 16 patients thus suggesting that neoadjuvant CAPTEM might improve the radicality of the surgical procedure (29).

The association of PRRT and chemotherapy has also been used as neoadjuvant therapy in pNENs, taking advantage from the radiosensitising effects of 5-FU. In this respect, the combination of PRRT with 177Lu-octreotate and 5FU chemotherapy was found to be effective in five nonfunctioning pancreatic and one duodenum NEN with inoperable disease, resulting in radiological response in all pNENs. Only one patient underwent surgery successfully after treatment and remained 12 months postoperatively alive and free of disease (28). Finally, combined PRRT and chemotherapy sandwiching two cycles of CAPTEM between two cycles of PRRT, has been proposed in neoadjuvant setting. This regimen resulted in favorable response rates with effective control of symptoms and longer PFS and OS in NEN patients with aggressive, both FDG- and SSTR-avid, metastatic progressive disease (30).

## Gastrointestinal Neuroendocrine Neoplasms

As for pNENs, neoadjuvant treatments have been proposed for other gastrointestinal NENS, data being mainly based on few case reports (**Table 1**). Neoadjuvant chemotherapy can be effective in patients with esophageal neuroendocrine carcinoma (ENEC), which are rare but aggressive neoplasms. In 2018, Yamamoto et al. reported the case of a patient with an ENEC who received neoadjuvant chemotherapy using etoposide and cisplatin. One course of chemotherapy led to tumor downstaging at endoscopy and to the absence of FDG accumulation at PET-CT examination. Seven weeks after chemotherapy, a thoracoscopic esophagectomy was performed and the histopathological examination of the resected specimen revealed no residual cancer cells, demonstrating a complete response with neoadjuvant treatment (31). Other few cases of ENECs treated with neoadjuvant chemotherapy have been reported (32–35). In three patients, cisplatin or combination chemotherapy caused an almost complete regression of the neoplasm with evidence of only microscopic foci of tumor in the resected esophageal specimen (32–34), while in other three cases treated with carboplatin/etoposide or combination chemotherapy a significant reduction in tumor burden was observed (35).

Neoadjuvant chemotherapy or chemoradiotherapy has been anecdotally reported to be effective also in rectal NENs (**Table 1**). In a study reporting on the management of patients with high grade rectum or anal canal neuroendocrine carcinomas, two cases were treated with preoperative pelvic chemoradiation. One of them received radiotherapy followed by consolidative cisplatin/5-FU, low anterior resection, and postoperative cisplatin/etoposide, while the second patient received induction oxaliplatin/irinotecan, followed by radiotherapy, trans anal excision, and additional oxaliplatin/irinotecan. Both patients had only microscopic foci of residual carcinoma at surgery (36). In another case report, a 50-year-old woman diagnosed with a liver mass and a G3 rectal NEN was treated with two cycles of neoadjuvant chemotherapy with etoposide and nedaplatin, this treatment being effective in rectal tumor but not liver metastasis shrinkage. Subsequently, the patient was switched to irinotecan plus nedaplatin, associated to octreotide LAR 30 mg/month because of neuroendocrine symptoms and MRI abdomen scan showed no significant changes in lesions size. Therefore, surgery was suggested and histopathological examination showed that the tumor downgraded from G3 to G2 thus suggesting that neoadjuvant chemotherapy may be effective in reducing primary lesion size and possibly grading, offering favourable conditions for less demolitive and more effective surgery.

The possible role of neoadjuvant PRRT and PRRT + chemotherapy combination was evaluated in small series of advanced gastrointestinal NENs (**Table 1**). Sowa-Staszczak et al. reported on neoadjuvant 90YDOTA-TATE treatment of five patients with foregut tumors, including three with pancreatic, and one with midgut NEN. According to RECIST criteria, disease stabilization was observed in four and partial responses in two patients, one with pancreatic and the second with the midgut NEN. In this latter case, tumor size decreased from 11 to 7.2 cm one month after PRRT. Five months later, a further reduction in tumor size was observed, enabling qualification for a laparotomy, which was performed 11 months after PRRT. However, only partial removal of the tumor was possible due to infiltration of the large vessels (15). In another study on 89 patients with disseminated and inoperable gastrointestinal NENs, Authors described one patient with a midgut tumor who was successfully treated with PRRT in a neoadjuvant setting, thus enabling an effective surgical intervention (37). The case of a 43-year-old man complaining of abdominal pain, vomiting, weight loss and flushes, who underwent CT examination that revealed upper and middle abdomen tumor was reported by Sowa-Staszczak et al. (38). Histopathological examination of tumor specimen obtained during exploratory laparotomy showed a well-differentiated NET according to the 2000 WHO classification. The patient received five cycles of chemotherapy (streptozocin and 5-FU) without any response and then he underwent PRRT with 90Y-DOTA-TATE. The subsequent CT scan revealed a reduction in tumor size and the patient was therefore candidate to a second laparotomy for a partial excision of the tumor. Then he was treated with long-acting SSA and two additional courses of 90Y-DOTA-TATE that induced a further reduction of tumor size, potentially enabling a further laparotomy for curative surgery (38). Barber et al. reported their experience with PRRT as neoadjuvant treatment in five patients with NENs, one of them being diagnosed with a locoregional recurrence of a duodenal tumor. The patient was treated with one cycle of 177 Lu-DOTATATE, with a partial scintigraphy and biochemical response (28). On the other hand, Frilling et al. reported the case of a patient with a small-bowel well differentiated NEN metastasised to the root of the mesentery, who underwent four cycles of neoadjuvant PRRT with 177Lu-DOTATATE. A following 68Ga-DOTATATE PET/CT demonstrated high tracer uptake in the mesenteric and aortocaval tumor foci with significantly higher SUV than pre-treatment imaging with no change in size of either the mesenteric or the aortocaval lesions. The patient then underwent a modified liver free multivisceral transplantation (39).

Overall, the few reported experiences would suggest that neoadjuvant chemotreatment can be a successful management strategy in esophageal NEN, while too little data are available about chemo/radio-treatment of other non-pancreatic gastrointestinal NENs in a neoadjuvant setting. However, PRRT seems represent an option in selected cases in this context.

## Thoracic Neuroendocrine Neoplasms

Lung neuroendocrine neoplasms represent approximately 20–30% of all NENs. Based on clinical, histological and molecular data, lung NENs are classified in two main categories well differentiated neuroendocrine tumors (carcinoids) and poorly differentiated neuroendocrine carcinomas (NECs). Furthermore, lung carcinoids (LC) are classified in typical (low grade) and atypical carcinoids (intermediate grade) and lung NECs in large-cells and small-cells carcinomas (LCNEC and SCLC, respectively). Only low-quality evidence guides the therapeutic management of LC, and everolimus is the only approved drugs. However currently used systemic therapeutic options include somatostatin analogues, alkylating- and oxaliplatin-based chemotherapies and PRRT. Few data are available on the efficacy of neoadjuvant treatment in thoracic NENs and all these data come from small series and case reports (**Table 2**).

**Table 2:** Neoadjuvant therapies in lung NENs.

Year	Article type	Lung NENs	Neoadjuvant treatment	Outcome	Reference
2009	case report	undefined	chemo	complete response and resection	(42)
2014	research article	atypical carcinoids	chemo	no response, worse prognosis	(43)
2004	research article	NEC	chemo	complete resection	(44)
2006	research article	LCNEC	chemo	longer OS (stage I)	(45)
2011	research article	LCNEC	chemo	longer OS (stage IB-IIA)	(46)
2010	research article	LCNEC	chemo	higher 5-year survival rate	(47)
2019	research article	LCNEC, SCLC	chemo	higher 5-year survival rate	(48)
2018	case report	LCNEC	chemo	downsizing of the tumor, complete resection	(49)
2019	case report	LCNEC	chemo	downsizing of the tumor, complete resection	(50)
2010	research article	LCNEC	chemo	worse 5-year OS	(51)
2015	research article	Thymus NENs	chemo or radio	no effects	(52)
2008	case report	Thymus NENs	chemo	downsizing of the tumor, complete resection	(53)

NEC, neuroendocrine carcinoma; LCNEC, large cell neuroendocrine carcinoma; SCLC, small cell neuroendocrine carcinoma; Chemo, chemotherapy; radio, radiotherapy; OS, overall survival.

Srirajaskanthan el al. reported two patients with lung NENs that received a preoperative chemotherapy with 5-FU, cisplatin and streptozotocin, that induced a good response with consequently a curative resection, both patients being disease free at 36 months after surgery (42). A multicentric study by Daddi et al. reported six of 247 patients with atypical carcinoids treated with neoadjuvant chemotherapy as an initial diagnosis of SCLC was performed on fine needle aspiration biopsy. Though no data on the results of neoadjuvant treatment were clearly shown, Authors found an association between adjuvant and neoadjuvant treatments and a worse prognosis. These data do not support the efficacy of neoadjuvant therapies in terms of complete regression of the metastatic disease. However, these treatments might be effective in alleviating clinical signs and symptoms (43). The same Authors reported five patients with poorly differentiated NECs who underwent to induction therapy and surgery without disease recurrence at 5 years, but no information was available on the chemotherapy regimens used (44).

More data are available on the role of neoadjuvant therapies in LCNEC. In the multicenter retrospective study by Veronesi et al., 15% of 144 patients who underwent surgical resection for LCNEC, received neoadjuvant chemotherapy (i.e., platin/etoposide, gemcitabine, vinorelbine and taxol). In this study no association was found between neoadjuvant chemotherapy and survival except for stage I patients in whom induction or post-operative chemotherapy tended to be associated to a longer OS (OS rate at 3 years 100% vs 58%) (45). Sarkaria et al. retrospectively analyzed 100 patients with LCNEC operated at Memorial Sloan-Kettering Cancer Center. Twenty-four patients received neo-adjuvant platinum-based chemotherapy and 68% showed a partial response and 31% were characterized by a stable disease. The correlation analysis did not show any association between OS and neoadjuvant or adjuvant chemotherapy. The authors also performed a subgroup analysis in patients with completely resected advanced stage (IB–IIIA) disease and, in these patients, neoadjuvant and adjuvant chemotherapy resulted in an improved OS (2 vs 7.4 years) and 5 years OS rate (37% vs 51%) (46). Saji et al. retrospectively confirmed a positive effect of perioperative chemotherapy on survival in 45 patients with LCNEC. In this study, seven patients received a neoadjuvant chemotherapy (cisplatin and paclitaxel in four and cisplatin/topotecan in three, respectively) thus leading to a statistically significant higher 5-year survival rate (87.5% vs 58%) (47). Similar results were obtained by Ogawa et al. retrospectively evaluating a series LCNEC and SCLC who underwent complete resection. Seventy patients (31 with LCNEC and 32 with SCLC) received perioperative platinum-based chemotherapy and a significant improvement of the 5-year OS rates was observed (74.5% vs. 34.7%). Multivariate analysis revealed that perioperative chemotherapy, sublobar resection, and lymph node metastasis were independently associated with survival (48). The efficacy of perioperative chemotherapy in patients with LCNEC has been further confirmed by some case reports (49, 50). In particular, Mauclet et al. reported a case of a 41-years old women with a large LCNEC with mediastinal involvement. After an ineffective first line chemotherapy with cisplatin etoposide, patient underwent to palliative radiotherapy and second line therapy with Nivolumab that led to a downsizing of the tumor. Patient underwent surgery with the complete removal of the tumor and histology showed an absence of viable tumor cells, while necrosis and fibrosis were observed (50). Finally, the retrospective analysis of 63 patients with LCNEC showed that neoadjuvant platin-etoposide based chemotherapy was associated with a trend towards a worse 5-year OS rate despite a partial response in 12 cases was observed. Authors suggested that the negative association between neoadjuvant chemotherapy and survival could be due to the fact that only patients with stage III tumors received induction chemotherapy (51).

NENs of the thymus are very rare tumors, accounting for 0.4% of all carcinoid tumors. Based on WHO 2015, also thymic NENs are classified in two main histopathological and clinical categories: well differentiated tumors, typical and atypical carcinoids and poorly differentiated tumors, small cell and large cell carcinoma. These tumors could be associated to ectopic hormonal secretion, in particular adrenocorticotropic hormone secretion or to multiple endocrine neoplasia type 1. The prognosis of patients with thymic NENs is poor because of the high incidence of local recurrence and distant metastasis and 5-year OS vary from 30–70%. As for other lung NENs, few data are available on neoadjuvat therapy in thymic NENs (**Table 2**). Few data are available on neoadjuvant therapy in thymic NENs. Filosso et al. reported 25 patients with primary thymic NENs treated by induction therapy (19 by chemotherapy and six by radiotherapy), these treatments having no impact on survival (52). Dham et al. described a clinical case of a 40-year-old man with unresectable typical carcinoid of the thymus. Three weeks of treatment with sunitinib 50 mg per day for 4 weeks with 2 weeks off and octreotide LAR 30 mg very 4 weeks, induced a tumor shrinkage that led to curative surgery of the mediastinal mass and no evidence of disease recurrence was evident 12 months after surgery (53).

## Conclusions

Data on neoadjuvant treatment of NEN patients are scanty, mainly based on inhomogeneous and often incomparable retrospective studies of limited numbers of patients and prospective studies are necessary to clarify the role of neoadjuvant therapy in this clinical setting. Available literature on pancreatic NEN patients suggests PRRT to be variably successful as a neoadjuvant approach, as well as chemotherapy to be more promising in patients with advanced synchronous pancreatic NELM, while—in the same setting—combined chemo/radio/PRRT-therapies would not be supported by sufficient evidence. Neoadjuvant PRRT, chemo or chemoradio-therapies have been anectodotally reported to be effective in non-pancreatic gastrointestinal NENs. Among thoracic NENs, neoadjuvant chemotherapy has been inconsistently reported to be beneficial in LCNC patients, also in relationship to disease stage, while little evidence would suggest neoadjuvant treatment to be negligible in thymic tumors. Therefore, it is advisable to use a neoadjuvant approach with caution, as the effects on quality of life and long-term results in terms of prolonged survival remain yet to be confirmed and the choice of this therapeutic approach should be discussed for each single patient in a multidisciplinary setting.

## Author Contributions

AL conceived and authored the final draft of the manuscript. FF, MR, and RM provided further content, added key references, and authored sections of the manuscript. AF and AC revised the manuscript. All authors contributed to the article and approved the submitted version.

## Conflict of Interest

The authors declare that the research was conducted in the absence of any commercial or financial relationships that could be construed as a potential conflict of interest.

## Publisher’s Note

All claims expressed in this article are solely those of the authors and do not necessarily represent those of their affiliated organizations, or those of the publisher, the editors and the reviewers. Any product that may be evaluated in this article, or claim that may be made by its manufacturer, is not guaranteed or endorsed by the publisher.
